# Cardiovascular Risk Among Patients With Moderate-to-Severe Plaque Psoriasis in Southern Sri Lanka: A Case-Control Study

**DOI:** 10.7759/cureus.85057

**Published:** 2025-05-29

**Authors:** Achala Liyanage, Gayani Liyanage, Binari Wijenayake, Bandujith Yapa, Malathi Pushparani, Vijitha de Silva, Shinichi Imafuku, Sarath Lekamwasam

**Affiliations:** 1 Dermatology, Faculty of Medicine, University of Ruhuna, Galle, LKA; 2 Pharmacology, Faculty of Medicine, University of Ruhuna, Galle, LKA; 3 Dermatology, Galle National Hospital, Galle, LKA; 4 Dermatology, District General Hospital - Matara, Matara, LKA; 5 Dermatology, District General Hospital - Hambantota, Hambantota, LKA; 6 Community Medicine, Faculty of Medicine, University of Ruhuna, Galle, LKA; 7 Dermatology, Fukuoka University, Fukuoka, JPN; 8 Medicine, Faculty of Medicine, University of Ruhuna, Galle, LKA

**Keywords:** ascvd, cardiovascular risk, framingham score, metabolic syndrome, psoriasis

## Abstract

Introduction: Psoriasis is a chronic inflammatory skin condition associated with increased cardiovascular (CV) risk and a higher prevalence of comorbidities. The study aimed to assess CV risk and contributory factors in patients with chronic plaque psoriasis (CPP).

Methods: This case-control study included 80 patients with CPP attending selected dermatology clinics in Southern Sri Lanka, along with 80 age- and sex-matched controls from their neighbourhood. Data on demographic and disease-related factors were collected using an interviewer-administered questionnaire from consecutive patients with moderate to severe psoriasis, as defined by body surface area (BSA) and the Psoriasis Area and Severity Index (PASI). Anthropometric measurements, blood glucose, and lipid assays were conducted for both groups. Comorbidity was assessed using the Charlson Comorbidity Index (CCI) while CV risk was estimated using the Framingham Risk Score (FRS) and the Atherosclerotic Cardiovascular Disease (ASCVD) 10-year risk score.

Results: Mean (standard deviation, or SD) age of patients with CPP (n=80) was 51 (11) years, with 41 being male. The corresponding values of controls were 50 (10) years, with 41 being male. The median (interquartile range, or IQR) duration of psoriasis was 10 (6-20) years, median (IQR) BSA was 11.3% (5.5-20.4%), and median (IQR) PASI was 6.7 (5.3-10.8). Patients with psoriasis had a higher median CCI value (1 (1-3) vs. 0 (1-2); U=1819, p < 0.001) and higher occurrence of dyslipidaemia (odds ratio (OR) 5.69; confidence interval (CI): 2.03-15.93) compared to controls. Patients with CPP had higher triglycerides (TG) and triglyceride-to-high-density lipoprotein (TG/HDL) ratios compared to controls (p < 0.05 for both). Although CV risk scores were higher among patients with CPP, they did not differ significantly from those of controls.

Conclusion: Patients with CPP have higher comorbidity and unadjusted lipid levels. This highlights the need to educate healthcare professionals to adopt a more holistic approach in evaluating patients with CPP.

## Introduction

Psoriasis is no longer considered a mere skin disease owing to the comorbidities and chronic vascular inflammation associated with the disease. Psoriasis is characterized by hyperproliferation and abnormal differentiation of keratinocytes, leading to the formation of thick, scaly plaques, and is linked with significant psychological and metabolic comorbidities as well as poor quality of life. Many studies have shown a higher incidence of cardiovascular (CV) events in psoriasis. Liu et al. in a recent meta-analysis showed that psoriasis is linked with adverse CV outcomes including myocardial infarction, stroke, thromboembolism, arrhythmia, and CV death [1].

The pathomechanism of psoriasis involves a complex interplay between genetic, environmental, and immunological factors, and they are probably responsible for both skin manifestations and comorbidities associated with psoriasis. The inflammatory cascade is initially triggered by the innate immune system and later driven by T helper (Th) cells, namely Th1 and Th17, dendritic cells, and keratinocytes. Recently, more emphasis has been given to the Th17 cells and the interleukin-23/Th17 (IL-23/Th17) axis as the central role in psoriasis pathogenesis [2]. In psoriasis, dendritic cells secrete interleukin-12 (IL-12) and IL-23, while Th1 cells secrete tumor necrosis factor-alpha (TNF-α) and interferon-gamma (IFN-γ), and Th17 cells secrete interleukin-17 (IL-17) and interleukin-22 (IL-22). Further recirculation of T cells from the skin to the blood can spread inflammation at a systemic level.

The pathogenesis of atherosclerosis plaque formation also shares similar immunological mechanisms [3]. Atherosclerosis is the main pathological cause of arteriosclerotic CV disease. Increased circulating cytokines in psoriasis cause endothelial activation and the formation of nascent plaque, whereas Th1 cell differentiation stimulates plaque growth, and Th17 cells stimulate intraplaque haemorrhage and neoangiogenesis [3]. Elevated intraplaque IL-17 levels have the potential to exacerbate the deterioration of the fibrous cap, ultimately resulting in plaque rupture. Thus, psoriasis might be an independent risk factor for the development of adverse CV outcomes.

Studies have shown that patients with psoriasis have a high prevalence of CV risk factors, including diabetes, hypertension, obesity, and smoking. Obesity is particularly prevalent among patients with psoriasis [4], while the risk of developing psoriasis, especially the severe form of the disease, is also higher in obese individuals [5]. Similar to obesity, metabolic syndrome (MS) is also associated with psoriasis. It is intuitive to think that the link between psoriasis and increased CV risk may vary geographically. Factors such as genetics, vitamin D status, physical activity, social habits, and diet can be considered as potential contributors to this variation. Studies have shown that psoriasis is often associated with hypovitaminosis D [6], reduced physical activity [7], and increased psychological stress [8], all of which are recognized CV risk factors.

The main objective of this study was to assess the overall CV risk in patients with chronic plaque psoriasis (CPP) in Sri Lanka, using a case-control design in a multicentre setting. The multiple CV risk outcome measures assessed included CV risk estimated using validated tools: the Framingham Risk Score (FRS) and the Atherosclerotic Cardiovascular Disease (ASCVD) 10-year risk calculator. Other outcome measures included the prevalence of contributory CV risk factors, such as dyslipidaemia, obesity, diabetes mellitus, and hypertension, among patients with CPP.

## Materials and methods

This case-control study included patients with CPP attending dermatology clinics of three main tertiary care centres, namely the National Hospital, Galle, and District General Hospitals at Matara and Hambantota, which are tertiary care referral centres in the three administrative districts in the Southern province of Sri Lanka. All three are state-run hospitals providing free health care to all people in the region. Southern province has a population of 2.7 million, which equals 12.2% of the country’s population, and the ethnic composition of the region is broadly similar to the national values. The region has a mixed weather pattern with nearly half of the region in the dry zone and the rest in the wet zone.

Consecutive patients fulfilling selection criteria were recruited using a non-probability sampling method. Those aged 18 years or older with clinician-diagnosed CPP, with the involvement of body surface area (BSA) greater than 3% or a Psoriasis Area and Severity Index (PASI) score exceeding 5, indicative of moderate to severe disease, were selected for the study [9]. Pregnant or lactating women and those with systemic inflammatory diseases or immunodeficiency states were excluded. Controls were selected from the neighbourhood of patients, within 100 meters, who were not blood relatives, and they were matched for age (±5 years) and gender. They were devoid of psoriasis or any other chronic cutaneous or systemic inflammatory diseases. Data were collected from consecutive eligible patients until the sample size was achieved. A sample size of 80 was determined for the odds ratio (OR) of 1.46 observed for CV deaths among patients with CPP and considering a disease prevalence of 5% and 95% confidence intervals (CIs).

Sociodemographic and disease-related data were collected using a pretested interviewer-administered questionnaire, and comorbidities were assessed using the Charlson Comorbidity Index (CCI). The CCI provides a simple and readily applicable method to combine comorbidities, and it is the most extensively studied method of estimating the risk of death from comorbid disease [10].

Body weight was measured to the nearest 0.1 kg without footwear, and height was recorded using a portable stadiometer (Weight Master International, Japan) to the nearest 0.5 cm. Obesity was determined according to the cut-off values described by Misra et al.'s consensus statement [11]; normal body mass index (BMI): 18.0-22.9 kg/m^2^, overweight: 23.0-24.9 kg/m^2^, obesity: >25 kg/m^2^. Waist circumference (WC) was measured using a non-stretchable flexible tape in a horizontal position, just above the iliac crest, at the end of normal expiration, with the subject standing erect and looking straight forward. The cut-off values were taken as for men: 90 cm and 80 cm for women [11].

MS was diagnosed based on the criteria stated in the consensus statement for the diagnosis of obesity and the MS for Asian Indians, which is similar to South Asian Modified National Cholesterol Education Program (SAM-NCEP) criteria [11]. The criteria listed include (1) glucose intolerance defined as fasting glucose ≥5.6mmol/L (100 mg/dL) or known treatment for diabetes; (2) increased WC or abdominal obesity (≥90 cm for men and ≥80cm for women); (3) raised triglyceride (TG) levels ≥ 1.7mmol/L (150 mg/dL); (4) reduced high-density lipoprotein (HDL) <1.03mmol/L (<40 mg/dL) for men and <1.30 mmol/L (<50 mg/dL) for women; and (5) elevated blood pressure (systolic blood pressure, SBP ≥ 130 mmHg, and/or diastolic blood pressure, DBP ≥85 mmHg). When a subject had three of the five criteria, including previously detected hypertension, high TG, low HDL, impaired fasting glucose, impaired glucose tolerance or type 2 diabetes mellitus (T2DM), and those on treatment for the above disorders, the diagnosis of MS was made.

The CV risk profile was calculated using the FRS to estimate a 10-year risk of development of heart disease [12]. Variables including age, gender, SBP, total cholesterol (TC), HDL, treatment for hypertension, and smoking status were used as predictors of the tool, and the CV risk was categorized as low (<10%), intermediate (10-20%) and high (>20%) accordingly [13]. The ASCVD 10-year risk is another commonly used tool to estimate an individual's risk of developing CV events within the next 10 years and has been used to assess CV risk among patients with psoriasis [14]. This score is calculated based on age, sex, race, cholesterol levels (total, HDL), SBP, smoking status, diabetes, and hypertension treatment. The study obtained ethical approval from the Institutional Ethical Review Committee (ref. no.: 15.02.2018:3.6) and is in accordance with the 1964 Helsinki declaration.

The descriptive data are given as either mean (standard deviation, or SD) or median (interquartile range, or IQR), depending on data distribution. The independent t-test was used to compare cases and controls after applying a log transformation to correct for skewed data. Logistic regression models were used to assess the associations between variables. Data were adjusted for BMI only, as age, gender distribution, and smoking habits were not significantly different between the two groups. The level of probability was considered 0.05.

## Results

The mean (SD) age of patients with psoriasis (n=80) was 51 (11) years, and 41 of them were males. The median (IQR) duration of psoriasis was 10 (6-20) years, and 17.5% (n=14) had a family history of psoriasis. In terms of disease severity, median (IQR) BSA was 11.3% (5.5-20.4%), and median (IQR) PASI was 6.7 (5.3-10.8).

The mean (SD) age of the control group (n=80) was 50 (10) years, and 41 of them were males. Cases and controls were similar with regards to the sociodemographic characteristics, except for the occupational category in which non-skilled workers were more among the cases (Table 1).

**Table 1:** Comparison of sociodemographic data between patients with chronic plaque psoriasis and matching controls p < 0.05 was considered statistically significant (Chi-square test).

Sociodemographic characteristics	Cases, n (%)	Control, n (%)	χ² (df)	p-value
Educational status				
Less than secondary school	9 (64.3)	5 (35.7)	1.25 (1)	0.26
>=secondary school	71 (48.6)	75 (51.4)		
Social class				
Skilled	33 (37.5)	55 (62.5)	12.22 (1)	<0.001
Non-skilled	47 (69.1)	25 (30.9)		
Smoking habits				
Current	9 (47.4)	10 (52.6)	0.06 (1)	0.81
None	71 (50.4)	70 (49.6)		

Although diabetes mellitus, hypertension, dyslipidemia, and established coronary artery disease showed a higher prevalence among cases compared to controls, only the prevalence of dyslipidemia was significantly different, with an OR of 5.69 (95% CI: 2.03-15.93) (Table 2).

**Table 2:** Comparison of comorbidity between patients with chronic plaque psoriasis and matching controls p < 0.05 was considered statistically significant (independent samples t-test). CAD: coronary artery disease

Condition	Cases	Controls	OR (95% CI)	P-value
	n=80	n=80		
	(n, %)	(n, %)		
Diabetes mellitus	16 (59.3)	11 (40.7)	1.56 (0.67-3.63)	0.29
Hypertension	22 (53.7)	19 (46.3)	1.21 (0.59-2.48)	0.58
Dyslipidemia	22 (81.5)	5 (18.5)	5.69 (2.03-15.93)	0.000
Established CAD	4 (57.1)	3 (42.9)	1.35 (0.29-6.24)	0.69
Metabolic syndrome	23 (39)	36 (61)	0.49 (0.26-0.95)	0.03

The prevalence of MS was lower among the cases compared to controls, with an OR of 0.49 (95% CI: 0.26-0.95). Among cases, 18 patients (22.5%) had no comorbidities, 43 patients (53.8%) had mild comorbidities, 16 patients (20%) had moderate comorbidities, and three patients (3.8%) belonged to the severe comorbidity category. In comparison, the control group consisted of 43 individuals (53.8%) with no comorbidities, 34 individuals (42.5%) with mild comorbidities, three individuals (3.8%) with moderate comorbidities, and none in the severe comorbidity category (Table 2). CPP patients with multimorbidity had a higher median CCI value compared to controls (1 (1-3) and 0 (1-2), U=1819, p < 0.001).

When the general linear model was applied, unadjusted TG and TG/HDL showed statistically significant differences between the groups (p=0.028 and p=0.025, respectively). However, after adjusting for BMI, these associations became non-significant (p=0.15 and p=0.16, respectively). Although the differences were not significant, the BMI-adjusted SBP, DBP, and LDL were higher among cases, compared to controls (Table 3).

**Table 3:** Comparison of anthropometric and biochemical measurements between patients with chronic plaque psoriasis and matching controls * p < 0.05 was considered statistically significant; F-statistics were used for regression analysis. BMI: body mass index; HDL: high-density lipoprotein; TG: triglycerides; LDL: low-density lipoprotein; SBP: systolic blood pressure; DBP: diastolic blood pressure

Variable	Cases (n = 80), Mean ± SD	Controls (n = 80), Mean ± SD	P-value	Crude Estimate F (p-value)	BMI-Adjusted Estimate F (p-value)
Weight (kg)	58.5 ± 10.5	63.7 ± 12.3	0.005	NA	NA
Height (cm)	157.5 ± 8.8	159 ± 9.6	0.27		
BMI (kg/m^2^)	23.57 ± 3.7	25.1 ± 4.1	0.016		
Waist circumference					
Male	89.5 ± 9.6	91.1 ± 9.6	0.45		
Female	85.5 ± 9.9	87.5 ± 10.8	0.41		
Waist-hip ratio					
Male	0.92 ± 0.04	0.93 ± 0.05	0.38		
Female	0.87 ± 0.05	0.89 ± 0.05	0.07		
Fasting blood sugar	102.9 ± 37.2	103.4 ± 38.2	0.94	0.038 (0.84)	1.987 (0.16)
Total cholesterol	197.2 ± 45.8	192.2 ± 41.7	0.47	0.191 (0.66)	2.120 (0.14)
Triglycerides	102.6 ± 35.3	121.9 ± 58.5	0.013	4.917 (0.02)*	1.998 (0.15)
HDL				0.016 (0.89)	3.391 (0.06)
Male	43.95 ± 9.93	41.39 ± 9.52	0.24		
Female	46.13 ± 9.6	48.08 ± 10.25	0.39		
LDL	132.2 ± 40.1	123.7 ± 37.0	0.16	1.314 (0.25)	1.366 (0.24)
TG/HDL	2.4 ± 0.98	3.0 ± 1.9	0.012	5.092 (0.02)*	1.994 (0.16)
SBP: Mean (SD)	134 ± 22	129 ± 16	0.12	2.825 (0.09)	0.717 (0.39)
DBP: Mean (SD)	81 ± 13	78 ± 9	0.13	3.236 (0.07)	2.356 (0.12)

The comparison of CV risk between patients with psoriasis and controls, using FRS and ASCVD risk assessment tools, revealed no statistically significant differences (Table 4). The 10-year risk calculated using the FRS was marginally higher in cases compared to controls. Similarly, the 10-year and lifetime ASCVD risk was also higher in cases compared to controls, but did not differ significantly (Table 4).

**Table 4:** Comparison of cardiovascular risk assessment tools between cases and controls p < 0.05 considered statistically significant (Independent sample median test) FRS: Framingham Risk Score; MI: myocardial infarction; ASCVD: atherosclerotic cardiovascular disease

Variable	Cases Median (IQR)	Control Median (IQR)	P-value
FRS			
10-year risk of MI or death	3.05 (1.1-7.92)	2.5 (0.82-7.9)	0.75
Average 10-year risk of MI or death	7.5 (3.0-10.0)	7 (3-10)	0.87
ASCVD			
10-year ASCVD risk-calculated (%)	6.7 (2.27-10.77)	4.2 (1.8-8.32)	0.056
Risk with optimal risk factors (%)	2.5 (0.97-5.2)	1.8 (0.87-3.2)	0.22
Lifetime ASCVD risk (calculated) (%)	46 (39-50)	39 (39-50)	0.34
Risk with optimal risk factors (%)	5 (5-8)	5 (5-8)	0.99

## Discussion

In this study, both groups, 80 patients with moderate to severe psoriasis and 80 age- and sex-matched controls, were similar in most sociodemographic factors, except that non-skilled workers were more common among the cases. Psoriasis patients had a higher burden of comorbidities compared to controls assessed using the CCI. However, CV comorbidities were largely comparable between the groups, except for the higher prevalence of dyslipidaemia seen among the psoriasis patients. The control group had a higher prevalence of MS, possibly due to the higher BMI seen among them. After adjusting for BMI, there were no significant differences in the anthropometric and biochemical measurements between the two groups. Additionally, CV risk estimates, including the FRS and ASCVD 10-year risk, did not show significant differences between cases and controls.

Although, in general, studies have demonstrated a higher comorbidity and an elevated CV risk profile among patients with CPP, some studies have shown conflicting results [15]. Numerous large-scale population studies [16] and meta-analyses based on cohort studies [1] have shown that patients with psoriasis have an increased risk of MS and CV events, independent of the severity of psoriasis [17]. These associations are thought to stem from the chronic systemic inflammation seen in psoriasis and current treatment modalities, which contribute to endothelial dysfunction and thereby accelerate the atherosclerosis process. However, some studies report no significant difference in CV outcomes between psoriasis patients and control groups, despite the higher prevalence of traditional CV risk factors seen among patients with psoriasis [18]. A recent meta-analysis by Liu et al. reviewed 31 population-based studies and a total of 27 studies that had adjusted outcome data for traditional CV risk factors, including age, gender, socioeconomic status, previous CV disease, smoking status, BMI, and TC. Of them, 11 studies did not show an increase in CV events [1], in some even after adjusting for the psoriasis severity, which was based on the mode of treatment received [19]. These contradictory findings may arise from differences in study design, patient populations, the disease severity assessment, and the methods used to evaluate CV risk. Additionally, variations in how comorbidities are diagnosed and reported, along with differences in follow-up duration, may contribute to the inconsistent results.

The observed differences in comorbidity and CV risk profiles among patients with CPP could be attributed to geographical variations. Environmental factors [20], lifestyle habits [21], healthcare access [22], and genetic predispositions [23] differ across regions, potentially influencing the prevalence and severity of comorbidities in psoriasis patients. The dietary patterns [24], myths, and beliefs on dermatoses in cultural and religious backgrounds [25] vary between populations, and all of which are critical determinants of metabolic and CV health. Genetic factors and ethnicity also play a role in the variability of CV risk [26]. Studies have suggested that certain genetic polymorphisms, prevalent in specific populations, may influence both psoriasis and atherosclerosis, affecting CV outcomes [27]. Moreover, access to healthcare and the availability of treatments for psoriasis and other comorbid conditions can also influence outcomes [28]. Therefore, there is a growing need for region-specific studies to better understand these patterns.

In our study, the patients did not have established CV disease, yet the higher burden of traditional risk factors, particularly TG, may increase CV risk over time. Numerous studies have identified a link between elevated plasma TG and increased CV risk in patients with psoriasis [29].

Limitations

The tools used to assess CV risk tend to underestimate the risk, particularly among South Asians and individuals with chronic inflammatory disorders such as psoriasis [30]. Further selection bias and limitations inherent in the cross-sectional study design may have partly contributed to the inconclusive results observed in the overall CV risk profile.

## Conclusions

Our study highlights the complex relationship between psoriasis and CV risk and emphasizing the need for long-term risk prediction tools to assess the risks, as clustering of risk factors is prevalent among these patients. Developing and validating region-specific CV risk assessment models is essential to improve risk stratification. Additionally, healthcare providers should adopt a comprehensive approach that includes regular CV risk screenings for patients with psoriasis, even in the absence of established CV disease. These efforts can facilitate the early identification of at-risk individuals and support more targeted and effective prevention strategies.
